# Structures of a putative ζ-class glutathione *S*-transferase from the pathogenic fungus *Coccidioides immitis*

**DOI:** 10.1107/S1744309111009493

**Published:** 2011-08-13

**Authors:** Thomas E. Edwards, Cassie M. Bryan, David J. Leibly, Shellie H. Dieterich, Jan Abendroth, Banumathi Sankaran, Dhileep Sivam, Bart L. Staker, Wesley C. Van Voorhis, Peter J. Myler, Lance J. Stewart

**Affiliations:** a Seattle Structural Genomics Center for Infectious Disease, USA; b Emerald BioStructures Inc., 7869 NE Day Road West, Bainbridge Island, WA 98110, USA; c Department of Medicine, Division of Allergy and Infectious Diseases, MS 356423, School of Medicine, University of Washington, Seattle, WA 98195-6423, USA; d Berkeley Center For Structural Biology, Ernest Orlando Lawrence Berkeley National Laboratory, 1 Cyclotron Road, Berkeley, CA 94720, USA; e Seattle Biomedical Research Institute, 307 Westlake Avenue North, Suite 500, Seattle, WA 98109, USA; f Departments of Global Health and Medical Education and Biomedical Informatics, University of Washington, Seattle, WA 98195, USA

**Keywords:** *Coccidioides immitis*, coccidioidomycosis, dichloroacetic acid, glutathione *S*-transferase, maleylacetoacetate isomerase, phenylalanine and tyrosine metabolism, thioredoxin-like fold, Valley Fever

## Abstract

*Coccidioides immitis* is a pathogenic fungus populating the southwestern United States and is a causative agent of coccidioidomycosis, sometimes referred to as Valley Fever. Although the genome of this fungus has been sequenced, many operons are not properly annotated. Crystal structures are presented for a putative uncharacterized protein that shares sequence similarity with ζ-class glutathione *S*-transferases (GSTs) in both apo and glutathione-bound forms. The apo structure reveals a nonsymmetric homodimer with each protomer comprising two subdomains: a C-terminal helical domain and an N-terminal thioredoxin-like domain that is common to all GSTs. Half-site binding is observed in the glutathione-bound form. Considerable movement of some components of the active site relative to the glutathione-free form was observed, indicating an induced-fit mechanism for cofactor binding. The sequence homology, structure and half-site occupancy imply that the protein is a ζ-class glutathione *S*-transferase, a maleylacetoacetate isomerase (MAAI).

## Abstract 2

The pathogenic fungus *C. immitis* causes coccidioidomycosis, a potentially fatal disease. Here, apo and glutathione-bound crystal structures of a previously uncharacterized protein from *C. immitis* that appears to be a ζ-class glutathione *S*-transferase are presented.

## 1. Introduction

Glutathione *S*-transferases (GSTs) are detoxifying enzymes that appear across a wide range of aerobic organisms, including bacteria, fungi, plants and mammals. This enzyme superfamily conjugates toxins with glutathione and the conjugate is then eliminated from the organism. Trimeric membrane-bound and dimeric cytosolic GSTs have been described, although they do not appear to share sequence or structural similarities (Hayes & Pulford, 1995 ▶). All cytosolic GSTs share a common fold (βαβαββα, related to thioredoxin) and they are divided into a number of different classes (α, μ, ω, π, θ, ζ *etc*.) based on sequence analysis (Snyder & Maddison, 1997 ▶). Crystal structures have been determined for all major classes of GSTs (Dirr *et al.*, 1994 ▶; Sheehan *et al.*, 2001 ▶). These structures reveal a G-site where glutathione binds and an H-site where the hydrophobic substrate binds. Many organisms contain multiple GST isoforms that recognize different or occasionally overlapping substrates (Mannervik *et al.*, 1992 ▶). Human π-class GSTs are overexpressed in many cancers and are thought to be potential drug targets (Federici *et al.*, 2009 ▶; Quesada-Soriano *et al.*, 2011 ▶). In plants, GSTs are often associated with herbicide resistance (Prade *et al.*, 1998 ▶). In insects such as the mosquito that may be a carrier of malaria, GSTs are associated with insecticide resistance (Oakley *et al.*, 2001 ▶).

The Seattle Structural Genomics Center for Infectious Disease (SSGCID) is a structural genomics consortium dedicated to protein structure elucidation from National Institute for Allergy and Infectious Diseases (NIAID) class A–C organisms, with an emphasis on underserved and re-emerging pathogens (Myler *et al.*, 2009 ▶). One major goal is to provide a blueprint for structure-guided drug design by determining the three-dimensional structures of potential drug targets (Van Voorhis *et al.*, 2009 ▶). One target organism is *Coccidioides immitis*, a dust-borne pathogenic fungus that, along with the closely related species *C. posadasii*, causes coccidioidomycoses (Hector & Laniado-Laborin, 2005 ▶). *C. immitis*-related coccidioidomycosis is also known as Valley Fever because the disease is highly endemic in the San Joaquin Valley in California, USA. In addition, coccidioido­mycosis is endemic in semiarid regions of the southwestern United States, Mexico and parts of South America (Hector & Laniado-Laborin, 2005 ▶). Many *C. immitis* infections resolve spontaneously, although the infection is sometimes fatal, especially in immuno­compromised patients or patients with disseminated and central nervous system illnesses (Deus Filho, 2009 ▶). Existing therapies are relatively ineffective and new therapeutic options are needed (Hector & Laniado-Laborin, 2005 ▶). The genome of *C. immitis* has been sequenced (Sharpton *et al.*, 2009 ▶), although many of the open reading frames have been annotated as putative uncharacterized proteins. At the start of the SSGCID project in September 2007, the Protein Data Bank (PDB) only contained structures of one *C. immitis* protein: a chitinase (Bortone *et al.*, 2002 ▶; Hollis *et al.*, 2000 ▶).

Here, we report the structural characterization of one *C. immitis* gene product originally annotated as a ‘putative uncharacterized protein’. This protein (UniProt accession code D2YW48; formerly Q1E7Z9) has sequence homology to ζ-class GSTs of known structure and 98% sequence identity to a *C. posadasii* putative maleylaceto­acetate isomerase (MAAI; UniProt entry C5PGS4; Sharpton *et al.*, 2009 ▶). We determined a 2.2 Å resolution apo crystal structure of the *C. immitis* D2YW48 gene product by molecular replacement using the mouse ζ-class GST (PDB entry 2cz2; E. Mizohata, S. Morita, Y. Kinoshita, K. Nagano, H. Uda, T. Uchikubo, M. Shirouzu & S. Yokoyama, unpublished work) as a search model. In addition, we obtained a 1.85 Å resolution crystal structure of the D2YW48 gene product bound to glutathione in one half-site. In combination, the genomic sequence-similarity data, the apo structural fold and the half-site glutathione binding imply that the D2YW48 gene product is likely to be a ζ-class GST.

## 2. Methods

### 2.1. Protein expression and purification

The 231-residue *C. immitis* hypothetical protein gene (UniProt accession code D2YW48, formerly Q1E7Z9; XP_001247543) was amplified from genomic DNA and cloned into an expression vector (pAVA0421) encoding an N-terminal histidine-affinity tag followed by the human rhinovirus 3C protease cleavage sequence (the entire tag sequence is MAHHHHHHMGTLEAQTQGPGS, followed by the 231-residue *C. immitis* GST) using ligation-independent cloning (Aslanidis & de Jong, 1990 ▶). The clone was transformed into *Escherichia coli* BL21 (DE3) R3 Rosetta cells. Starter cultures of LB broth with appropriate antibiotics were grown for ∼18 h at 310 K. ZYP-5052 auto-induction medium was prepared as described by Studier (2005 ▶). The protein was expressed in a LEX bioreactor in the presence of antibiotics in 2 l sterilized auto-induction medium inoculated with the overnight starter culture. After 24 h at 298 K the temperature was reduced to 288 K for a further 60 h. The sample was centrifuged at 4000*g* for 20 min at 277 K. The cell paste was flash-frozen in liquid nitrogen and stored at 193 K. The frozen cells were resuspended in 25 m*M* HEPES pH 7.0, 500 m*M* NaCl, 5%(*v*/*v*) glycerol, 0.5% CHAPS, 30 m*M* imidazole, 10 m*M* MgCl_2_, 1 m*M* TCEP, 250 µg ml^−1^ AEBSF and 0.025%(*w*/*v*) azide at 277 K. Lysis was achieved by sonication followed by incubation with Benzonase (20 µl at 25 units per microlitre). Insoluble proteins and other cellular components were removed by centrifugation at 14 000 rev min^−1^ for 75 min at 277 K. The soluble fraction was then loaded onto an Ni–NTA His-Trap FF 5 ml column (GE Healthcare). The column was washed with 20 column volumes of wash buffer [25 m*M* HEPES pH 7.0, 500 m*M* NaCl, 5%(*v*/*v*) glycerol, 30 m*M* imidazole, 1 m*M* TCEP and 0.025%(*w*/*v*) azide]. The bound protein was eluted with seven column volumes of elution buffer [25 m*M* HEPES pH 7.0, 500 m*M* NaCl, 5%(*v*/*v*) glycerol, 1 m*M* TCEP, 250 m*M* imidazole and 0.025%(*w*/*v*) azide]. Cleavage of the N-terminal His tag was accomplished by dialysis with His-MBP-3C protease at 277 K overnight in 25 m*M* HEPES pH 7.5, 500 m*M* NaCl, 5%(*v*/*v*) glycerol, 1 m*M* TCEP and 0.025%(*w*/*v*) azide. The cleaved protein was recovered in the flowthrough and wash fractions of a subtractive Ni^2+^-affinity chromatography step that removed His-MBP-3C protease, uncleaved protein and the cleaved His tag. The collected cleaved protein (sequence GPGS followed by the 231-residue *C. immitis* GST) was loaded onto a HiLoad 26/60 Superdex 75 prep-grade column (GE Healthcare) equilibrated in 25 m*M* HEPES pH 7.0, 500 m*M* NaCl, 5%(*v*/*v*) glycerol, 2 m*M* dithiothreitol and 0.025%(*w*/*v*) azide. SDS–PAGE was used to determine fractions to pool. The purified protein was concentrated to 23.9 mg ml^−1^ and stored at 193 K.

### 2.2. Crystallization

Crystallization trials were set up according to a rational crystallization approach (Newman *et al.*, 2005 ▶) using the JCSG+ and PACT sparse-matrix screens from Emerald BioSystems and Molecular Dimensions. In addition, crystallization trials were set up with the Index and Crystal Screen HT sparse-matrix screens from Hampton Research. 0.4 µl protein solution at 23.9 mg ml^−1^ in 25 m*M* HEPES pH 7.0, 0.5 *M* NaCl, 5%(*v*/*v*) glycerol, 2 m*M* dithiothreitol and 0.025%(*w*/*v*) azide and an equal volume of precipitant were equilibrated against 80 µl reservoir solution at 289 K in sitting-drop vapor-diffusion format in 96-well Compact Jr plates from Emerald Bio­Systems. Within a week, apo crystals grew in the presence of 0.2 *M* lithium sulfate, 0.1 *M* Tris pH 8.5 and 30% PEG 4000 (Crystal Screen HT condition No. 17). Another protein sample was incubated with 10 m*M* reduced glutathione and crystallization trials were set up as above. Crystals grew within a week in the presence of 0.2 *M* ammonium sulfate and 30% PEG 8000 (Crystal Screen HT condition No. 30).

### 2.3. Data collection and structure determination

Both apo and glutathione-containing protein crystals were harvested, cryoprotected with a solution consisting of the precipitant supplemented with 25% ethylene glycol and vitrified in liquid nitrogen. A data set was collected at 100 K from an apo crystal under a stream of liquid nitrogen on the Advanced Light Source (ALS) beamline 5.0.2 as part of the ALS Collaborative Crystallography program (Table 1 ▶). A data set was collected from a glutathione-containing crystal at 100 K under a stream of liquid nitrogen using a Rigaku FR-E^+^ SuperBright X-ray generator with Osmic VariMax optics and a Saturn 944+ detector (Table 1 ▶). Data were reduced with *XDS*/*XSCALE* (Kabsch, 2010 ▶). The apo structure (Table 2 ▶) was solved by molecular replacement using the mouse ζ-class GST monomer (PDB entry 2cz2; E. Mizohata, S. Morita, Y. Kinoshita, K. Nagano, H. Uda, T. Uchikubo, M. Shirouzu & S. Yokoyama, unpublished work) as a search model in *Phaser* (McCoy *et al.*, 2007 ▶) from the *CCP*4 suite (Winn *et al.*, 2011 ▶). The structure was rebuilt using *ARP*/*wARP* (Langer *et al.*, 2008 ▶). The glutathione-bound structure (Table 2 ▶) was solved by molecular replacement using the apo structure as a search model in *Phaser* (McCoy *et al.*, 2007 ▶). The final models were obtained after numerous rounds of refinement in *REFMAC* (Murshudov *et al.*, 1997 ▶) and manual rebuilding in *Coot* (Emsley & Cowtan, 2004 ▶). Structures were assessed for correctness and validated using *MolProbity* (Davis *et al.*, 2007 ▶; Chen *et al.*, 2010 ▶).

## 3. Results and discussion

### 3.1. Apo structure

**Figure 1:**
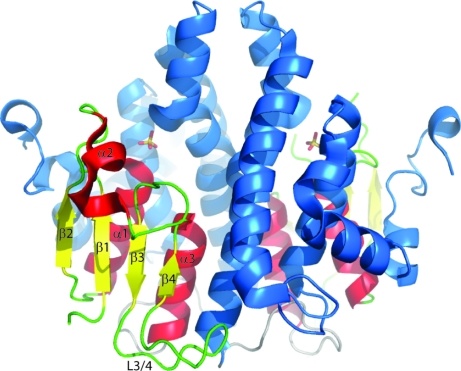
The 2.2 Å resolution apo crystal structure of a previously uncharacterized protein and putative ζ-class GST from *C. immitis* shown in cartoon representation. The thioredoxin-like domain (βαβαββα) is shown with α-helices in red, β-sheets in yellow and loops in green. The α-helical domain is shown in blue. The sulfate ion in the H-site is shown in stick representation. All figures were prepared with *PyMOL* (DeLano, 2002 ▶).

The putative uncharacterized protein from *C. immitis* (UniProt ID D2YW48) investigated here contains 231 residues. The 2.2 Å resolution apo structure contains two pseudosymmetrically related dimers in the asymmetric unit. Each protomer consists of an N-­terminal βαβαββα thioredoxin-like fold common to all GSTs as well as a C-­terminal α-helical domain (Fig. 1 ▶). The *C. immitis* protein exhibits a nonsymmetrical dimer in which one protomer has several more ordered residues than the other; residues 39–52 are dis­ordered in the second protomer. These residues correspond to the cap of the active site and coordinate glutathione in the G-site (see below).

All four chains in the asymmetric unit contain a sulfate molecule that binds to the backbone N atom and side-chain hydroxyl of Ser15 as well as the side chain of Gln124 and forms water-mediated interactions with several additional residues. A sulfate ion is observed in approximately the same orientation off Ser15 as in the H-site of the human ζ-class GST crystal structure (Polekhina *et al.*, 2001 ▶) and is believed to simulate the expected binding of the substrate dichloro­acetic acid (Ricci *et al.*, 2004 ▶). In the human GSTZ1-1 crystal structure the sulfate ion is also coordinated by Arg175. The equivalent Arg190 in the *C. immitis* structure resides in a different rotomer conformation without forming interactions with the sulfate ion (which is approximately 4.8 Å away in each protomer); it is involved in salt-bridge interactions within the crystal lattice.

### 3.2. Glutathione-bound structure

**Figure 2:**
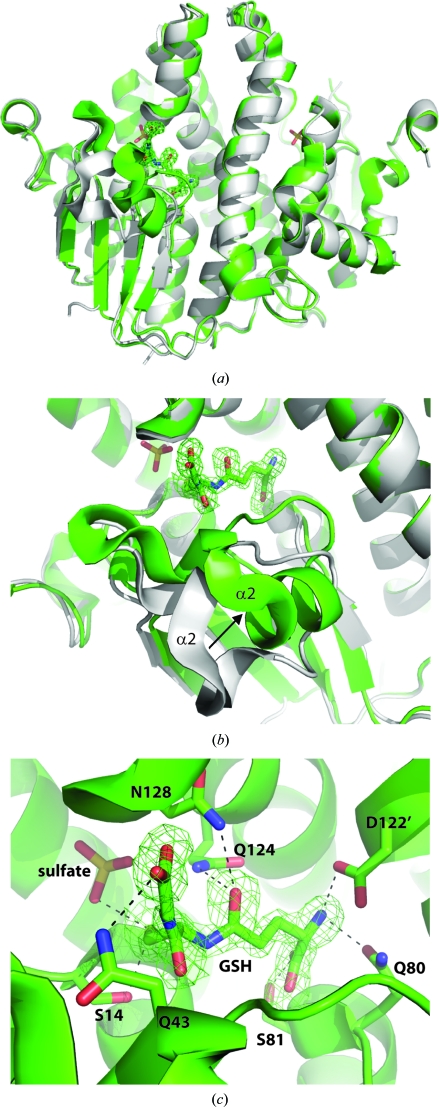
(*a*) Overlay of the 2.2 Å resolution apo crystal structure of a putative ζ-class GST from *C. immitis* in gray with a 1.85 Å resolution glutathione-bound structure in green. Glutathione and sulfate ions are shown in stick representation. An unbiased OMIT map (|*F*_o_| − |*F*_c_|) is shown in green mesh contoured at 2.5σ. (*b*) Close-up of the overlay shown in (*a*) showing the large-scale movement of α2 and loops upon glutathione binding. (*c*) Interactions of glutathione (GSH) with G-site residues. All residues other than Asp122′ are from the same protomer.

A 1.85 Å resolution crystal structure was determined from a crystal grown in the presence of 10 m*M* glutathione (∼10.7 equivalents relative to protein at 0.93 m*M*). The structure was of a different crystal form to the apo structure and contained one dimer in the asymmetric unit. This structure revealed the presence of glutathione in one protomer, again revealing a nonsymmetrical dimer (Fig. 2 ▶). The position of glutathione is almost identical to that observed in human GSTZ1-1 (Polekhina *et al.*, 2001 ▶) and forms interactions with the conserved residues Ser14, Gln43, Gln80, Ser81, Asp122′ (from the other protomer), Gln124 and Asn128 (Fig. 2 ▶ *c*). The distance between the Cys16 S atom and the glutathione S atom was 3.7 Å. Similarly, the distance between these atoms was 2.8 Å in the human GSTZ1-1 crystal structure, indicating the lack of a disulfide bond (Polekhina *et al.*, 2001 ▶). A sulfate ion was observed in the same position off Ser14 as in the apo crystal structure, again with Arg190 positioned away from the sulfate ion (4.6 Å). The glutathione-bound protomer was ordered from residues 3 to 230, whereas the other unliganded protomer was ordered from residues 3 to 39 and residues 55 to 231. Furthermore, α2 and the residues that surround the glutathione moved significantly relative to the apo structure (r.m.s.d. of 0.77 Å for all residues, but in the range 1.9–7.0 Å for residues 42–56; Fig. 2 ▶ *b*), which is indicative of an induced-fit mechanism for cofactor binding.

### 3.3. Comparison with other GSTs

**Figure 3:**
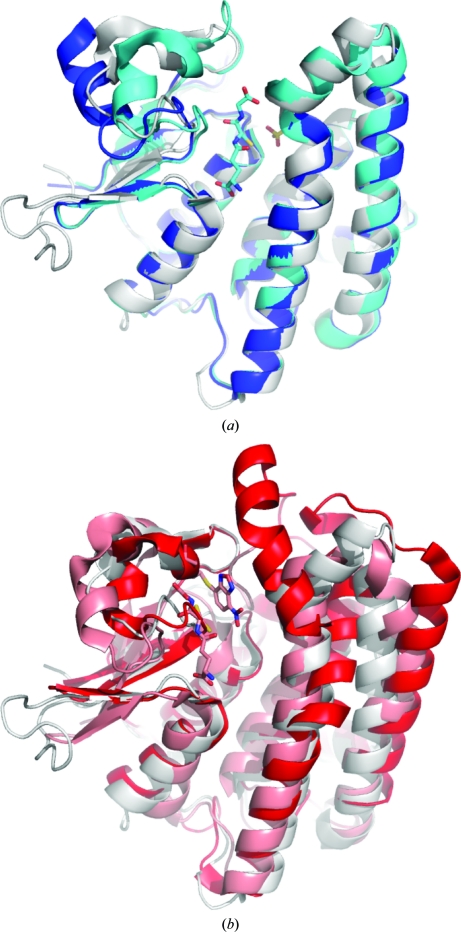
(*a*) Overlay of the crystal structures of a putative ζ-class GST from *C. immitis* shown in gray with human ζ-class GST in light blue (with glutathione and sulfate ion shown as sticks; Polekhina *et al.*, 2001 ▶) and mouse ζ-class GST in dark blue (Mizohata *et al.*, unpublished work). (*b*) Overlay of the crystal structures of a putative ζ- class GST from *C. immitis* shown in gray with human π-class GSTP1-1 in pink [with GST and the inhibitor 6-(7-nitro-2,1,3-benzoxadiazol-4-ylthio)hexanol (NBDHEX) in stick representation; Federici *et al.*, 2009 ▶] and human θ-class GSTT1-1 in dark red (Tars *et al.*, 2006 ▶).

The *C. immitis* protein investigated here shares sequence homo­logy with several ζ-class GSTs of known structure, specifically human (*Homo sapiens*, 44% sequence identity; Polekhina *et al.*, 2001 ▶), mouse (*Mus musculus*, 44% sequence identity; E. Mizohata, S. Morita, Y. Kinoshita, K. Nagano, H. Uda, T. Uchikubo, M. Shirouzu & S. Yokoyama, unpublished work), a plant (*Arabidopsis thaliana*, 36% sequence identity; Thom *et al.*, 2001 ▶) and a bacterium (*Ralstonia* sp., 35% sequence identity; Marsh *et al.*, 2008 ▶). The *C. immitis* structure overlays remarkably well with the human GSTZ1-1 (r.m.s.d. 0.95 Å), mouse GSTZ (r.m.s.d. 1.01 Å), *A. thaliana* GSTZ (r.m.s.d. 1.42 Å) and *Ralstonia* GSTZ (r.m.s.d. 1.06 Å) structures (Fig. 3 ▶ *a*); superposition calculations were performed using the *CCP*4 suite (Winn *et al.*, 2011 ▶). Despite clearly sharing the same thioredoxin-like fold (Fig. 3 ▶ *b*), the *C. immitis* structure has such weak sequence homology to other classes of GST (typically <10% over the full protein sequence) that its structure overlays poorly with these classes. For example, human π GST 1-1 (PDB entry 3gus; Federici *et al.*, 2009 ▶) overlays with an r.m.s.d. of 2.53 Å, human θ GST 1-1 (PDB entry 2c3q; Tars *et al.*, 2006 ▶) overlays with an r.m.s.d. of 2.22 Å and human κ GST (PDB entry 1yxz; Li *et al.*, 2005 ▶) overlays with an r.m.s.d. of 2.93 Å.

In the *C. immitis* structure glutathione is bound in only one half-site. This is consistent with binding studies on human GSTZ1-1, which showed half-site glutathione binding even at saturating glutathione concentrations and are suggestive of a cooperative mechanism (Ricci *et al.*, 2004 ▶). Interestingly, the crystal structure of human GSTZ1-1 bound to glutathione (Polekhina *et al.*, 2001 ▶) contains only one molecule in the asymmetric unit, with the other protomer being generated by crystallographic symmetry, indicating that the two protomers are identical. Similarly, the crystal structures of the *Ralstonia* ζ-class GST (Marsh *et al.*, 2008 ▶) show glutathione or a glutathione conjugate bound in both protomers. Thus, it appears that the crystal structures and solution-based binding studies of ζ-­class GSTs are not always in agreement on half-site or full-site glutathione binding.

The *C. immitis* crystal structure contains an asparagine-rich and serine-rich loop between β3 and β4 (residues 64–74) that is not present in other ζ-class GST crystal structures (Figs. 1 ▶ and 3 ▶). This loop interacts almost exclusively with the other protomer and therefore may stabilize dimer formation or be involved in recognition of another protein or some other as yet undiscovered function.

**Figure 4:**
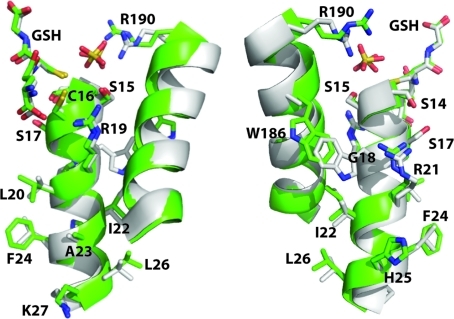
Comparison of α1 structural and sequence conservation in the putative ζ-class GST from *C. immitis*, shown in green, and human ζ-class GST, shown in gray (Polekhina *et al.*, 2001 ▶). The two orientations are shown about 180° apart. In each structure glutathione (GSH) and the sulfate ion are shown in stick representation.

One of the defining characteristics of ζ-class GSTs is the high sequence conservation of α1, which contains the sequence SSCS(H/W)RVRAIL (Board *et al.*, 1997 ▶). The equivalent residues of the *C. immitis* protein contain three single point differences (SSCS**G**R­**L**RAI**F**) yet retain a highly similar backbone structure (Fig. 4 ▶). Two of these are conservative differences. The nonconserved difference at residue 18 is the presence of a glycine residue where there is normally a histidine or tryptophan residue. However, the *C. immitis* structure reveals a tryptophan residue at position 186 that fills the pocket generated by the lack of a side chain at Gly18; the equivalent position in human or mouse GSTZ1-1 is Ala171 (Fig. 4 ▶). Thus, the *C. immitis* protein contains a compensating amino-acid change that allows it to retain a highly similar α1 structure.

## 4. Conclusions

ζ-Class GSTs have been identified across a wide range of organisms (Board & Anders, 2005 ▶; Board *et al.*, 1997 ▶). Here, we report two crystal structures of a putative uncharacterized protein from *C. immitis*, the pathogenic fungus that causes coccidioidomycosis. Based on sequence homology, structural similarity, half-site glutathione binding and sulfate ion H-site binding, the protein investigated here is likely to be a ζ-class GST/MAAI. The next step is to determine the actual enzymatic activity of the protein and identify its substrates. To address this, we plan to investigate the binding of this *C. immitis* GST to a wide variety of commercially available glutathione conjugates as well as dichloroacetate, which has been described as a suicide inhibitor of GSTZ/MAAI (Ricci *et al.*, 2004 ▶; Stacpoole, 2011 ▶). Such investigations will be reported in due course.

## Supplementary Material

PDB reference: CoimA.00410.a, 3lg6

PDB reference: 3n5o

## Figures and Tables

**Table 1:** Data-collection statistics Values in parentheses are for the highest of 20 resolution shells.

Ligand	Apo	Glutathione
Space group	*P*2_1_2_1_2_1_	*C*222_1_
Unit-cell parameters (Å)	*a* = 51.7, *b* = 99.6, *c* = 184.4	*a* = 49.6, *b* = 110.9, *c* = 168.5
Wavelength (Å)	1.000	1.5418
Resolution range (Å)	50–2.20 (2.24–2.20)	50–1.85 (1.90–1.85)
No. of unique reflections	49300	39710
Multiplicity	6.8 (6.0)	5.5 (1.9)
Completeness (%)	99.7 (97.9)	98.8 (88.5)
*R*_merge_†	0.121 (0.529)	0.072 (0.359)
Mean *I*/σ(*I*)	13.2 (4.8)	17.8 (2.3)

† *R*_merge_ = 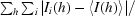 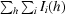.

**Table 2:** Refinement and model statistics Values in parentheses are for the highest of 20 resolution shells.

Ligand	Apo	Glutathione
Resolution range (Å)	50–2.20 (2.26–2.20)	50–1.85 (1.90–1.85)
*R*_cryst_†	0.173 (0.252)	0.160 (0.247)
*R*_free_†	0.226 (0.285)	0.205 (0.306)
R.m.s.d. bonds (Å)	0.015	0.015
R.m.s.d. angles (°)	1.463	1.377
Protein atoms	6845	3409
Hetero atoms	20	35
Waters	590	460
Mean *B* factor (Å^2^)	23.4	15.9
Glutathione *B* factor (Å^2^)	—	20.0
Residues in favored region (%)	99.1	99.5
Residues in allowed region (%)	99.1	100
*MolProbity*‡ score [percentile]	1.23 [100th]	1.24 [99th]
PDB code	3lg6	3n5o

† *R*_free_ = 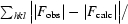 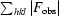. The free *R* factor was calculated using 5% of the reflections, which were omitted from the refinement (Winn *et al.*, 2011 ▶).

‡ Chen *et al.* (2010 ▶), Davis *et al.* (2007 ▶).
